# Adequate union rates for the treatment of acute proximal fifth metatarsal fractures

**DOI:** 10.1007/s00167-020-06072-8

**Published:** 2020-05-30

**Authors:** Q. G. H. Rikken, J. Dahmen, N. C. Hagemeijer, I. N. Sierevelt, G. M. M. J. Kerkhoffs, C. W. DiGiovanni

**Affiliations:** 1 Foot and Ankle Research and Innovation Laboratory, Department of Orthopaedic Surgery, Massachusetts General Hospital, Harvard Medical School, Boston, USA; 2 Department of Orthopaedic Surgery, Amsterdam Movement Sciences, Amsterdam UMC, University of Amsterdam, Meibergdreef 9, Amsterdam, The Netherlands; 3 Academic Center for Evidence Based Sports Medicine (ACES), Amsterdam, The Netherlands; 4 Amsterdam Collaboration for Health and Safety in Sports (ACHSS), International Olympic Committee (IOC) Research Center Amsterdam UMC, Amsterdam, The Netherlands; 5 Foot and Ankle Service, Department of Orthopaedic Surgery, Massachusetts General Hospital, Newton-Wellesley Hospital, Harvard Medical School, Boston, MA USA; 6 Specialized Center of Orthopedic Research and Education (SCORE), Xpert Ortopedie, Amsterdam, The Netherlands

**Keywords:** Fifth metatarsal, Fracture, Conservative, Surgery

## Abstract

**Purpose:**

To compare the bone healing, clinical, and return to daily activity outcomes after either surgical or conservative management of acute zone 1, 2, and 3 fifth metatarsal fractures.

**Methods:**

A literature search was performed to identify studies published from the earliest record to January 2019 using EMBASE (Ovid), MEDLINE via PubMed, CINAHL, and Web of Science. All articles assessing clinical outcomes of acute proximal fifth metatarsal fractures were included. Bone healing and clinical outcomes were thereafter calculated using a simplified pooling method.

**Results:**

Thirty-two articles comprising of a total of 1,239 fractures were included, of which one was a randomized controlled trial, seven were prospective studies, and 24 were retrospective studies. 627 zone 1 fractures demonstrated union rates of 93.2% following conservative treatment and 95.1% following surgical treatment. Conservatively managed zone 1 fractures were displaced 49.5% of the time, compared to a rate of 92.8% for the surgically treated cases. For Jones’ (zone 2) fractures, bone healing outcomes of conservative versus surgical treatment showed union rates of 77.4% versus 96.3%, refracture rates of 2.4% versus 2.1%, and mean time to union of 11.0 weeks versus 9.4 weeks, respectively. Only ten proximal diaphyseal (zone 3) fractures were reported, with a mean return to work of 8.2 weeks.

**Conclusion:**

Acute zone 1 fractures are preferably treated conservatively as similar union rates were found after both conservative and surgical management. In contradistinction, acute zone 2 fractures demonstrate higher union rates and faster time to union when treated surgically. The outcomes of acute zone 3 fractures are rarely reported in the literature, so treatment recommendations remain unclear. Further research of proximal fifth metatarsal fractures is warranted to provide more definitive conclusions, but current findings can aid surgeons during the shared clinical decision making process.

**Level of evidence:**

IV.

**Electronic supplementary material:**

The online version of this article (10.1007/s00167-020-06072-8) contains supplementary material, which is available to authorized users.

## Introduction

Acute fractures of the proximal fifth metatarsal are a common injury of the foot [14, 20, 39]. Currently, the choice between surgical or conservative treatment of these fractures is primarily based on anatomical location and degree of fracture displacement. Fractures of the proximal fifth metatarsal have been subdivided into three regions, as described by Lawrence and Botte [27]: tuberosity avulsion fractures (zone 1), Jones’ fractures (zone 2) and, proximal diaphyseal fractures (zone 3). Even though controversy persists, the current trend in orthopaedic care has been to treat non-displaced zone 1 fractures primarily conservatively, to treat significantly displaced zone 1 fractures surgically, and to treat zone 2 and 3 fractures either conservatively or surgically depending on overall patient expectation and activity level [21, 23, 50, 55]**.** The first aim of this review was therefore to assess available literature to determine the bone healing outcomes following both surgical and conservative treatment of each anatomical zone of acute proximal fifth metatarsal fractures. A secondary aim of this study was to evaluate the clinical and return to activity related outcomes of each treatment strategy. The clinical utility of this work is aimed at providing better guidance for clinicians and patients during the shared decision making process.

## Material and methods

The preferred reporting items for systematic reviews and meta-analyses statement (PRISMA) was used as a guideline for the present study [29]. The study protocol was prospectively registered in the PROSPERO registry for systematic reviews with registry number: CRD42019122682.

### Search strategy

Studies from the earliest record (June 1902), until January 2019 were retrieved from EMBASE (Ovid), MEDLINE via PubMed, CINAHL, and Web of Science. The search strategy can be reviewed in the supplementary materials 2.

### Eligibility criteria and study selection

**Fig. 1:**
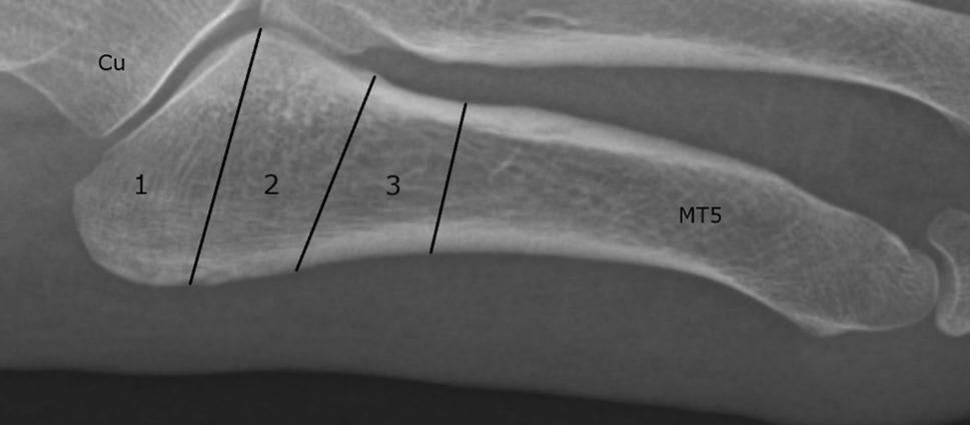
Lawrence and Botte classification for proximal fifth metatarsal (MT5) fractures. zone 1: tuberosity avulsion fractures (1), zone 2: Jones’ fractures (2), zone 3: proximal diaphyseal fractures (3)

All randomized controlled trials (RCTs), controlled non-randomized trials, prospective-, and retrospective cohorts*,* comparative studies and case series that investigated conservative and/or surgical treatment of the acute proximal fifth metatarsal fracture types as proposed by Lawrence and Botte (see Fig. 1) were included. Case series were only included if they included 10 or more participants per treatment or fracture group. Furthermore, papers written in English, French, German and Dutch were eligible for inclusion. The exclusion criteria are listed in Table 1. Fracture acuteness was defined by each article’s respective author. Additionally, Torg 1 fractures were included as acute fractures [22, 28]. No patient age, demographic or publication date restrictions were applied. Moreover, backward citation searching was used to find additional eligible articles. Authors were contacted by email if results were unclear for multiple anatomical locations or if results were combined between acute and stress fractures to enable separated datasets. If no response was recorded following two reminder emails, the respective author’s paper was thereafter excluded*.* Two authors (Q.R. and J.D.) independently screened titles and abstracts, and full-text articles with predetermined inclusion and exclusion criteria as stated above, using Covidence (https://www.covidence.org/home). Disagreement was resolved by an attempt to reach consensus. If no consensus was reached a third reviewer (N.H.) was decisive.

**Table 1:** Exclusion criteria

Reoperation or non-union as primary treatment
No separate data for acute fractures per zone available
Stress fractures as primary fracture included or acute fracture data not separable
Review- and animal studies
Follow-up less than 12 weeks

### Methodological quality

Methodological quality was assessed by two independent reviewers (Q.R. and J.D.). Included articles were screened for bias using the Methodological Index for Non-Randomized Studies (MINORS) [46]. When no consensus was reached on the MINORS score, a third author (N.H.) was decisive.

### Data extraction

A standardized form was used to collect study characteristics and outcome measures. Data was only extracted if outcomes could be identifiable based on fracture type and/or anatomical location. Study characteristics retrieved included number of patients and fractures, age, gender, fracture location and type, mean follow-up time, percentage of physically active patients/athletes and study design. Bone healing outcomes included number of unions, number of combined delayed- and non-unions, number of refractures, time to union and the percentage of displaced tuberosity avulsion fractures (> 2 mm). Healing- and non-healing rates were calculated as the percentage of radiographical (non/delayed)- unions, refractures of the total number of fractures per treatment modality. Refractures were, therefore, not considered as a healing/treatment complication but an independent event. Time to return to activities and time to return to work were extracted as the same outcome variable representing a general return time to daily functioning. At last, all possible clinical outcome- or patient reported outcome measures were collected.

### Statistical and data analysis

Heterogeneity of the included studies was assessed with an *I*^2^ statistic and the results per study were visualized by means of a forest plot (eye-ball test) [16]. Due to high heterogeneity in study design it was decided that a formal meta-analysis was not possible. Therefore, a simplified pooling method was used to combine data from included studies for quantitative analysis. Pooled means and proportions were calculated by weighting the number of fractures per study for each specific zone or treatment modality (i.e. the bone healing rate of all surgically treated zone 2 fractures weighted by the number of fractures per individual study). The results for acute fractures were analysed for each Lawrence and Botte zone. Time units were converted either to weeks or months, depending on the outcome variable. A subgroup analysis of specific treatment modality was performed to assess the effect of independent treatments. No comparative synthesis was performed, as the indication to perform surgery is different and dependent on multiple, sometimes underreported, factors such as fracture displacement [6, 7, 26]. Ranges of reported pooled means encompassed the lowest and highest mean values from the included studies. Median values were transformed to mean values according to the formula from Hozo et al. [17]. Data analysis was carried out using Stata 15 (StataCorp LP, College Station, TX).

## Results

### Article selection

**Fig. 2:**
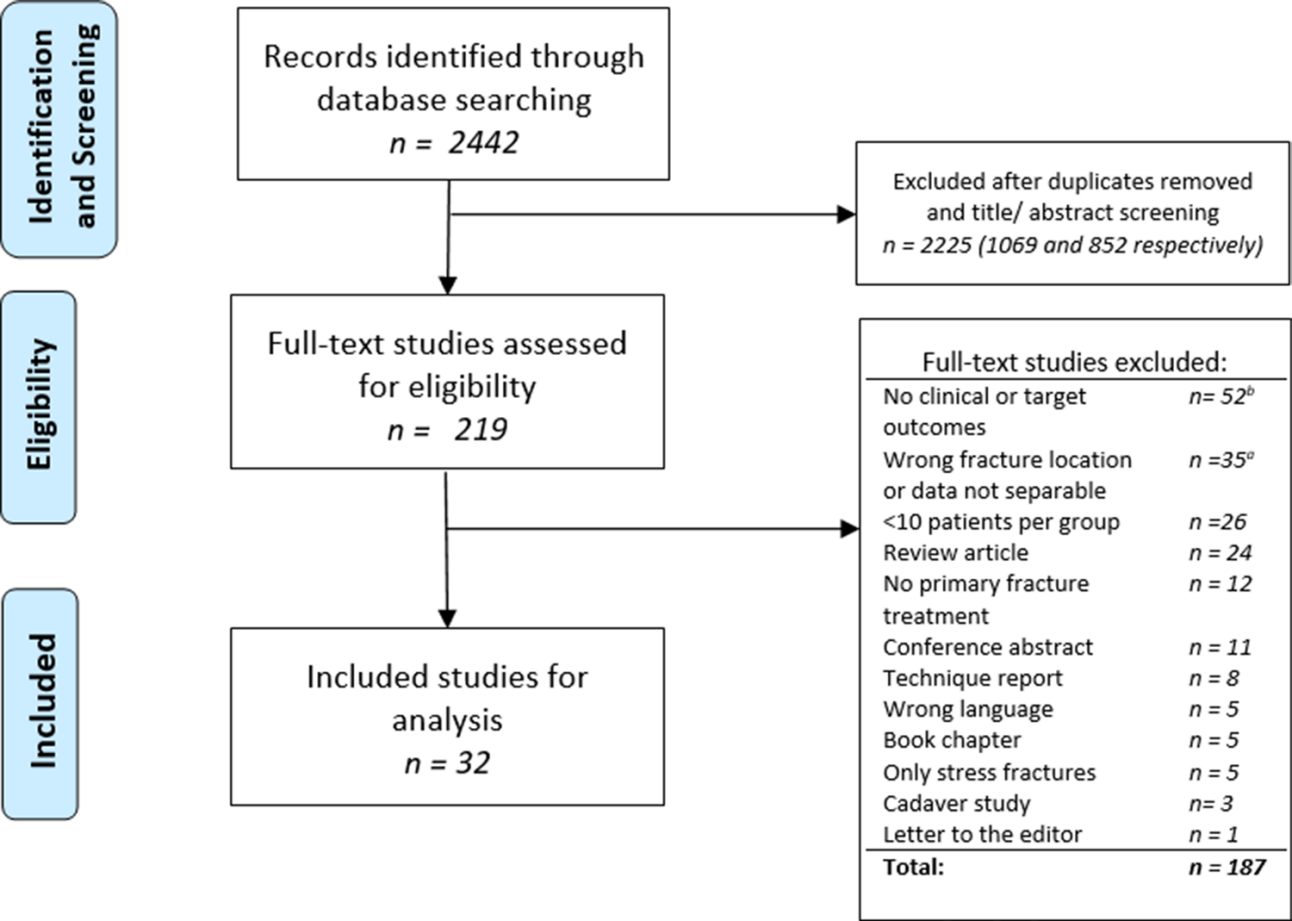
PRISMA flowchart of study selection

After screening, consensus was reached in all cases for article selection. The literature search yielded 2442 articles. After title and abstract screening and removal of duplicates, 217 articles were eligible for full-text screening and then 32 full articles were included for final analysis. Reasons for exclusion are listed in Fig. 2. One RCT, three prospective comparative (PC) studies, four prospective case series (PCS), five retrospective comparative studies (RC) and 19 retrospective case series (RCS) were included. Furthermore, one author provided additional data [37]. A total of 1239 proximal fifth metatarsal fractures were included in this review, with a mean age of 39.2 years. As reported in the supplementary materials 3, nine different clinical outcome scores were reported in the included studies, of which the American Orthopaedic Foot & Ankle Society (AOFAS) midfoot score [24] was most frequently reported and used for analysis.

## Methodological quality

Consensus was reached between the reviewers regarding grading of methodological quality. Non-comparative studies scored an average of 7.2 points out of 16 (range: 3–12). Comparative studies had an average MINORS score of 15.4 out of 24 (range: 11–21). The individual MINORS score per study is displayed in supplementary materials 4.

## Outcome measures

### Tuberosity avulsion fractures (zone 1):

For zone 1, 711 fractures were included from 19 studies [1, 3, 4, 6, 11–13, 15, 23, 25, 26, 32, 37, 45, 50, 52–54, 58], of which 531 (74.7%) fractures were managed conservatively and 180 (25.3%) were managed surgically. Conservatively treated fractures were non-displaced in 267 (50.3%) cases, displaced in 77 (14.5%) cases, and unrecorded for 187 (35.1%) cases. Surgically treated fractures were displaced in 167 (92.8%) and non-displaced in 13 (7.2%). An overview of the pooled patient characteristics and pooled treatment outcomes is shown in Table 2. Overall, conservative treatment showed a lower pooled bone healing rate and pooled mean time to union compared to surgical treatment of 91.0% versus 96.1% and 8.5 weeks versus 7.6 weeks, respectively (Table 2). Pooled results from secondary outcome measures can be seen in Table 2. The effect of displacement on bone healing outcome showed that displaced tuberosity avulsion fractures had similar pooled bone union rates when treated surgically and conservatively (Table 3).

**Table 2:** Zone 1 pooled results

Treatment		Mean age, years (range)	Mean FU, months (range)	Primary outcomes					Secondary outcomes	
				Union, *n* (%)	Delayed/non-union, *n* (%)	Refracture, *n* (%)	Total (*n*)	Mean time to union, weeks (range)	Mean time to activities, weeks (range)	Mean AOFAS (range)
Surgery	Overall	42.4 (range: 25.6–50)	19.3 (range: 12–37.7)	173 (96.1%)	7 (3.9%)	0 (0%)	180	7.6 (*n* = 106) (range: 1.6–9.6)	11.0 (*n* = 91) (range: 9.1–12.5)	93.3 (*n* = 159) (range: 87.8–98.2)
	IMS^a^	36.8 (25.6–47.0)	12.2 (12.0–13.0)	58 (95.1%)	3 (4.9%)	–	61	8.9 (*n* = 49) (7.7–9.6)	10.5 (*n* = 46) (8.1–12.5)	90.3 (*n* = 70) (88.1–97.7)
	Plate	44.0 (34.4–50.0)	13.6 (12.0–17.0)	58 (96.7%)	2 (3.3%)	–	60	7.0 (*n* = 60) (5.9–8.8)	11.5 (*n* = 45) (10.4–12.0)	94.6 (*n* = 60) (93.2–98.2)
	BIS	47.2 N/A	5.3 N/A	6 (100%)	0 (0%)	–	6	5.3 (*n* = 6) N/A	–	93.5 (*n* = 6) N/A
	K-wire	48.4 N/A	30.6 N/A	31 (96.9%)	1 (3.1%)	–	32	–	–	96.5 (*n* = 32) N/A
	Unknown	43.7 N/A	37.7 N/A	20 (95.2%)	1 (4.8%)	–	21	–	–	-
Conservative	Overall	39.2 (range: 12.4–51.5)	13.9 (range: 2.8–32.4)	334 (91.0%)	33 (9.0%)	0 (0%)	367	8.5 (*n* = 155) (range: 6.1- 16.1)	7.1 (*n* = 269) (range: 2.1- 17.4)	92.8 (n = 159) (range: 87- 96.4)
	Cast	36.9 (12.4–51.5)	9.5 (8.6–10.4)	137 (93.2%)	10 (6.8%)	–	147	6.14 (*n* = 75) N/A	7.3 (*n* = 72) (2.8–16.1)	90.7 (n = 62) (87.0–94.0)
	Shoe/boot	43.3 (40.0–44.5)	13.8 (2.8–22.0)	42 (84%)	8 (16%)	–	50	–	4.6 (*n* = 148) (2.1–6.7)	96.4 (n = 59) N/A
	Bandage	39.9 (36.0–47.5)	2.8 N/A	60 (96.8%)	2 (3.2%)	–	62	6.7 (*n* = 45) (6.4–7.1)	–	90.5 (n = 17) N/A
	Combined	38.3 N/A	32.4 N/A	25 (71.4%)	10 (28.6%)	–	35	16.1 (*n* = 35) N/A	17.4 (*n* = 35) N/A	-
	Unknown	37.7 (23.0–37.9)	25.0 N/A	70 (95.9%)	3 (4.1%)	–	73	–	–	-

*IMS* intra-medullary screw, *BIS* bicortical screw, *N* number, *N/A* not applicable

**Table 3:** Pooled weighted bone union outcomes displaced and non-displaced fractures zone 1

		Union	Non/delayed-union	Total	Time to union (weeks), range
ND	S	–	–	–	–
	C	33 (100%)	0 (0%)	33	–
D	S	92 (94.8%)	5 (5.2%)	97	8.3 (5.3–9.6)
	C	48 (92.3%)	4 (7.7%)	52	–

Only included studies with separate data for displaced or non-displaced fractures

*S* surgery, *C* conservative, *D* displaced (> 2 mm), *ND* non-displaced (< 2 mm)

### Jones’ fractures (zone 2)

All pooled results for zone 2 fractures are listed in Table 4. 518 zone 2 fractures were included from 21 studies [1, 3–7, 12, 19, 22, 25, 30, 32, 34, 36, 37, 41, 44, 49–51, 56], of which 318 (61.4%) fractures received conservative treatment and 200 (38.6%) received surgical treatment. The pooled patient characteristics and pooled treatment outcomes of zone 2 fractures are presented in Table 4. Pooled bone healing outcomes of conservative versus surgical treatment showed union rates of 77.4% versus 96.3%, refracture rates of 2.4% versus 2.1%, and mean time to union of 11.0 weeks versus 9.4 weeks, respectively. Pooled secondary outcomes are displayed in Table 4.

**Table 4:** Zone 2 pooled results

Treatment		Mean age, years (range)	Mean FU, months (range)	Primary outcomes					Secondary outcomes	
				Union, *n* (%)	Delayed/non-union, *n* (%)	Refracture, *n* (%)	Total (*n*)	Mean time to union, weeks (range)	Mean time to activities, weeks (range)	Mean AOFAS (range)
Surgery	Overall	34.0 (range: 23–54.7)	26.7 (range: 17–46)	193 (96.5%)	7 (3.5%)	4 (2.0%)	200	9.4 (*n* = 149) (range: 5.7–27.4)	7.7 (*n* = 29) (range: 5.8–10.7)	92.5 (*n* = 28) (range: 90.0—94.2)
	IMS^a^	31.4 (30.1–32.7)	25.8 (24.6–27.0)	132 (95.7%)	6 (4.3%)	1 (0.7%)	138	10.2 (*n* = 97) (8.5–11.9)	5.8 (*n* = 18) N/A	-
	Plate	46 N/A	17.0 N/A	11( 100%)	0 (0%)	–	11	8.1 (*n* = 46) N/A	10.7 (*n* = 46) N/A	90.0 (*n* = 46) N/A
	BIS	43.2 N/A	N/A	17 (100%)	0 (0%)	–	17	6.6 (*n* = 17) N/A	–	94.2 (*n* = 17) N/A
	Ex-Fix	25.2 N/A	46 N/A	9 (90%)	1 (10%)	1 (10%)	10	6.5 (*n* = 10) N/A	–	–
	Unknown	36.2 (29.8–42.6)	29.5 N/A	24 (100%)	0 (0%)	2 (8.3%)	14	11.0 (*n* = 14) N/A	–	–
Conservative	Overall	40.4 (range: 18.6–59.4)	40.9 (range: 3–186)	213 (81.9%)	47 (18.1%)	6 (2.3%)	260	11.0 (*n* = 157) (range: 7.3–26.5)	6.2 (*n* = 85) (range: 2.7–15.2)	95.5 (*n* = 67) (range: 92.5–97.5)
	Cast	30.5 (18.6–45.0)	26.3 (15.0–40.2)	48 (75%)	12 (25%)	2 (3.1%)	64	16.5 (*n* = 50) (7.4–26.5)	5.1 (*n* = 25) (2.7–6.7)	97.5 (*n* = 25) N/A
	Shoe/boot	40.3 (18.6–50.8)	24.3 (15.0–40.2)	13 (37.1%)	22 (62.9%)	–	35	8.0 (*n* = 35) N/A	4.9 (*n* = 33) (2.9–6.8)	92.5 (*n* = 17) N/A
	Bandage	46.1 (43.3–53.0)	152 (16.1–186)	34 (89.5%)	4 (10.5%)	2 (5.3%)	38	7.3 (*n* = 38) N/A	–	–
	Combined	39 (35.0–48.6)	3.0 N/A	66 (89.2%)	8 (10.8%)	–	74	8.6 (*n* = 71) (7.3–15.2)	15.2 (*n* = 10) N/A	95.6 (*n* = 25) N/A
	Unknown	57.3 (23.0–59.4)	27.8 N/A	52 (100%)	0 (0%)	2 (3.8%)	52	12.1 (*n* = 3) N/A	–	–

*IMS* intra-medullary screw, *BIS* bicortical screw, *N* number, *N/A* not applicable

### Proximal diaphyseal fractures (zone 3)

Ten acute zone 3 fractures from a single case series were found through the literature search (also see supplementary materials 1), with the patients having a mean age of 57 years and follow-up of 15 months (range 12–24) [4]. Individual study characteristics can be viewed in the supplementary materials 1. In this series all patients were treated conservatively and no bone healing outcomes were presented. Six patients were treated with casting and reported a return to work at mean 8.0 weeks and a AOFAS score of 85. The functional (shoe) group, consisting of four patients, returned to work at mean time of 8.5 weeks and had a mean AOFAS score of 83.

## Discussion

The most notable finding of this study was that union rates for tuberosity avulsion fractures were found to be independent of both initial treatment choice and degree of fracture displacement. Non-displaced zone 1 fractures demonstrated particularly uncomplicated union rates following non-operative management. Outcome analysis of displaced zone 1 fractures, however, also demonstrated similar union rates for surgical and conservative treatment, albeit with a more limited dataset. In contradistinction, both higher union rates and faster times to union were found following the surgical management of zone 2 fractures when compared to conservative treatment methods. Acute proximal diaphyseal (zone 3) fractures appear to be a rare occurrence in the literature, and therefore an optimized treatment algorithm cannot be determined at this time.

### Tuberosity avulsion (zone 1) fractures

When disregarding fracture displacement, zone 1 fractures were found to have a lower pooled healing rate when treated conservatively. However, multiple factors contribute to this finding. First, fracture displacement is an important treatment indication for zone 1 fractures as displaced fractures tend to be fixed surgically. Naturally, the healing rate of non-displaced fractures might differ, as treatment indications could vary among different orthopaedic institutions, which may also influence the healing rate of conservative treatment. Secondly, the averaged low overall bone healing rate following conservative treatment in this review may in part be attributable to the low healing rate (71.4%) of a single study included [25]. Possible factors affecting bone union in this series could be the high percentage of displaced fractures, the relatively high number of older patients, and a lack of standardization amongst treatment regimens. Thirdly, this review pooled the outcomes of both athletes and non-athletes, the former of whom were more likely to have been treated surgically and thus introduced possible selection bias [12, 26, 53]. Most zone 1 fractures were non-displaced and managed conservatively in both athletes and non-athletes, however, with high union rates and few complications that are consistent with current literature [42, 43, 50, 52]. Non-displaced tuberosity avulsion fractures should therefore be treated conservatively. This is in line with a systematic review article by Kerkhoffs et al. who reviewed the literature in 2012 and similarly found favourable healing rates and return to activities after conservative treatment [21].

For displaced zone 1 fractures the pooled mean healing rate was found not to be clinically different. It should be noted, however, that union data for this analysis was only available for a small number of patients, resulting in a limited assessment. Therefore, these results should be interpreted with caution and comparative research is warranted as authors have cited fracture displacement of more than 2 mm as a reason for surgical intervention with excellent results [6, 11, 23, 26, 32, 53, 54, 58]. This can partly be explained by the difficulty of stable anatomical reduction of the avulsion fragment without fixation due to tendinous traction of the peroneus brevis, peroneus tertius, and lateral plantar aponeurosis [48]. A single study by Wu et al. included in this systematic review directly compared surgical and conservative therapy, favouring surgery for return to activities and AOFAS scores [53]. Unfortunately, however, none of the primary outcomes of the present review were assessed in the aforementioned study.

### Jones’ (zone 2) fractures

No clear consensus exists regarding optimal treatment of zone 2 fractures [21]. Traditionally, surgical treatment was primarily advised for athletes who desired faster return to sport, but this has now been increasingly advised for the non-athletic population as well [36, 41]. While the present review did not stratify between athletes and non-athletes, an overall higher union rate and faster time to union were identified with the surgically treated group.

Jones’ fractures are prone to stress forces and have a poor blood supply leading to an impaired healing tendency [8, 47]. Ekstrand et al. found no clear correlation between the Torg classification and the injury circumstances (i.e. injury due to trauma or overuse) [12]. Ekstrand concluded that at primary presentation most fractures were registered as traumatic, even though half were found to be stress related per radiograph. Certain fractures that presented as acute may have occurred based on underlying stress phenomena, thereby incorrectly suggesting an acute fracture. Conceivably, a subset of the acute zone 2 fractures in this systematic review did involve a repetitive component but were considered acute due to their clinical presentation. This may have negatively affected the pooled union rate of conservative treatment, however, because stress fractures have been observed to have higher union rates and shorter union times when treated surgically [7, 12, 31, 33]. An example of this is the included RCT in this review which randomized allocated active military staff to either the casting or intra-medullary screw (IMS) treatment group [36]. Screw-fixation showed a significantly higher bone healing rate and a shorter time to union than casting. Additionally, early surgical intervention resulted in a lower incidence of non-unions, delayed unions and refractures as well as a faster return to activities. It should be noted, however, that the military population remains at inherent risk of repetitive microtrauma as part of the pathoaetiology of their fracture outcome. Further research is necessary to stratify between the genuinely acute, sub-acute (where there is a potential stress riser component), and chronic stress related fractures. Additionally, biomechanical studies are warranted to further elaborate on the processes underlying sub-acute and stress fractures.

Surgical management for the acute zone 2 fractures seems justified when considering the superior bone healing outcomes. Treatment providers, however, should consider inherent complications related to surgical procedures [7, 9, 36, 41]. Surgical treatment may become the preferred choice of treatment depending on patient factors such as age, BMI, social- and work status and patient choice. Even though outcomes seem less favourable after conservative treatment, its non-invasiveness and the fact that there is low quality evidence supporting surgical treatment makes it justifiable as treatment for zone 2 fractures, especially in cases where there is no suspicion for repetitive trauma.

### Proximal tuberosity fractures

Zone 3 fractures are generally considered stress fractures that can be treated both surgically and conservatively [7, 10, 25, 35, 38, 40]. Only one case series of acute fractures was found with comparable outcomes of conservative treatment for zone 1 and 2 [4]. However, case reports by Zelko et al. [57] and Arangio et al. [2] reported varying results of their primarily surgically treated patients. Traditionally, the “Jones fracture” occurs in both zone 2 and 3, as described in the original 1902 article [18]. Conventional orthopaedic literature describes a Jones fracture as a fracture at the proximal diaphysis and metaphysis junction of the fifth metatarsal without distal extension beyond the fourth to fifth intermetatarsal articulation [7, 27, 36]. The varying fractures termed “Jones fractures” could potentially explain the underrepresentation of acute zone 3 fractures in the literature. Acute zone 3 fractures are rarely reported in the literature, prompting a call for more research into these fractures.

### Methodological considerations

The outcomes of this systematic review must be interpreted within the context of its design. First, few high-level articles were included in this study as was shown by the low MINORS score of the body of included articles. Therefore, interpretation of the results in the present systematic review must be done with caution. Additionally, only one zone 1 study, and three zone 2 studies directly compared surgery with conservative therapy, making a formal statistical comparison by meta-analysis methodologically unfit. A simplified pooling technique was therefore used, allowing a large body of articles to be included which could introduce bias as a result of including varying study designs and patients populations. Performing a comparative synthesis for the present study was deliberately avoided because treatment indications for these fractures differed within this body of literature. Therefore, it should be noted that the clinical outcomes of this study have to be interpreted with caution and should not be used for treatment decisions for individual patients.

Second, this review only included acute fractures of the proximal fifth metatarsal, as stress fractures have an inherent tendency for impaired healing. There seems, however, to be no uniform definition that clearly stratifies acute- from stress fractures. This might have had an effect on the results of this study as it might have been possible that patients that actually had a stress fracture were included in this systematic review, thus biasing results.

## Conclusion

Non-displaced acute tuberosity (zone 1) avulsion fractures are preferably treated conservatively given comparable union rates and clinical outcomes were found, regardless of chosen management, and fracture healing is typically uncomplicated. Ideal management of displaced zone 1 fractures remains unclear due to the small number of cases identified, although available evidence suggests that both conservative and surgical treatment demonstrate comparable union rates. Acute zone 2 fractures were found to have higher union rates and faster time to union when managed surgically, which the current literature supports as the treatment of choice for all such injuries. Acute zone 3 fractures continue to be rarely reported in the literature, so treatment outcomes remain insufficiently documented and thus preclude any formal management recommendation. Higher level of evidence research on proximal fifth metatarsal fractures will be necessary to provide more definitive conclusions.

## Electronic supplementary material

Below is the link to the electronic supplementary material.

Supplementary file1 (PDF 381 kb)

## Data Availability

Not applicable.
