# Supplementary material for: Adequate union rates for the treatment of acute proximal fifth metatarsal fractures
Source: Knee Surg Sports Traumatol Arthrosc. 2020 May 30;29(4):1284–93. doi: 10.1007/s00167-020-06072-8 (PMC7973408; doi:10.1007/s00167-020-06072-8)
Supplement: Supplementary file 1 — Supplementary file1 (PDF 381 kb) [file 167_2020_6072_MOESM1_ESM.pdf]

Supplementary materials 1: individual study table

Study	Design	Zone	Treatment	No. patients	No. Fractures	Male/ female	Mean Age (yrs)	Mean FU (months)	Bone healing outcomes					Mean time return to activities/ work	Mean AOFA-score/ PROMS	Total % athletes
									Union (n, %)	Delayed/non-union (n, %)	Refracture (n, %)	Time to union (wks)	Displacement in % (>2mm)			
Baumbach *et al* 2017[4]	RCS	Zone 1	C: Shoe, WB	**Total:** 39<br>23	23	**Total:**17/22	**Total:**<br>40 years (SD: 15)	**Total:**<br>22 months (SD: 10, range: 6-40)					26%	2.1 weeks (SD: 1.4)	VAS: 97 (SD: 4)	-
		Zone 2	C: Shoe, WB	16	16								38%	2.9 weeks (SD: 2.1)	VAS: 95 (SD: 4)	
Biz *et al* 2018[5]	RC	Zone 1	C: Cast	**Total:** 154<br>43	43	**Total:**60/94	44.6 years	**Total:**<br>15 months (range: 12-24)					0%	7.2 weeks	Midfoot score: 94	34%
			C: Functional (shoe)	59	59		44.5 years							6.7 weeks	96.4	
		Zone 2	C: Cast	25	25		45 years							6.7 weeks	97.5	
			C: Functional	17	17									6.8 weeks	92.5	
		Zone 3	C: Cast	6	6		57 years							8.0 weeks	85	
			C: Functional	4	4									8.5 weeks	83	
Chee-kid *et al* 2009[6]	RCS	Zone 2	C: back-slab, functional bandaging	25	25	10/15	40.9 years (range 19 - 71)	3 months	24 (96%)	1 (4%)	-	7.8 weeks (SD: 1.7, range: 6-12)	38%		95.6 (SD: 7.7)	16%
Choi *et al* 2013[7]	PCS	Zone 1	S: plate	**Total:** 17<br>6	6		**Total:**<br>46 years (SD: 16, range: 19 - 77)	**Total:**<br>17 months (SD: 1, range: 12-28)	6 (100%)	0 (0%)	-	7.1 weeks (SD: 1.6, range: 5.4- 9.7)	100%	10.4 weeks (SD: 2, range: 8.6-14)	93.0 (SD: 7) range (85-100)	Unknown
		Zone 2	S: plate	11	11				11 (100%)	0 (0%)	-	8.1 weeks (SD: 1.4. range: 6-10.6)	0%	10.7 weeks (SD: 1, range: 9.3-12)	90.0 (SD: 6) (82-100)	
Chuckpaiwong *et al* 2008[8]	RC	Zone 2	C: shoe /cast, WB	**Total:** 61	17	24/8	27 years (SD: 11.1)	**Total:** 40 months (SD: 11.7 months, range: 24 – 72)	14 (82.4%)	3 (17.6%)	-	26.5 weeks (SD: 0.7)	0%	2.7 weeks (SD: 0.7)		27.9% high-level, 57.4% recreational
			S: U		18				16 (88.9%) including 3 non-unions from c group	2 (11.1%)	-	27.4 weeks (SD: 32.2)	-	5.8 weeks (SD 12.8)		
Egol *et al* 2007[12]	PCS	Zone 1	C: shoe	49	50	8/41	41.9 years (range: 17 - 81)	12 months	42 (83%)	8 (17%)	-	-	32%	3.1 weeks		Unknown

Study	Design	Zone	Treatment	No. patients	No. Fractures	Male/ female	Mean Age (yrs)	Mean FU (months)	Bone healing outcomes					Mean time return to activities/ work	Mean AOFA-score/ PROMS	Total % athletes
									**Union (n, %)**	**Delayed/non-union (n, %)**		**Time to union (wks)**	**Displacement in %** (>2mm)			
Ekstrand *et al* 2013[13]	PCS	Zone 1	C: U	**Total:** 38<br>1	**Total:** 38	**Total:** 38/0	**Total:** 23 years (SD: 3, range 18 - 33)		1 (100%)	0 (0%)	-	-	-			100%
		Zone 2	S: U	14					14 (100%)	0 (0%)	2 (14%)	11 weeks	-			
			C: U	3					3 (100%)	0 (0%)	2 (66%)	12.1 weeks	-			
Gray *et al* 2008[14]	RC	Zone 1	C: Plaster	**Total:** 37<br>20	20	8/12	46 years (range: 16 - 81)	**Total:** 2.8 months	18 (90%)	2 (10%)	-	-	-		90.5[a] (range: 82- 100)	Unknown
			C: bandage	17	17	7/10	40 years (range: 15 -82)		15 (88.2%)	2 (11.8%)	-	-	-		87.5[a] (range 70- 100)	
Herrera-soto *et al* 2007[16]	RCS	Zone 1	C: Cast	44	45	20/24	12.4 years (range: 9 - 19)		40 (88.9%)	5 (11.1%)	-	6.14 week	32.1%			Unknown
Josefsson *et al* 1994[19]	RCS	Zone 2	C: bandage; 2 none	24	24	15/9	43.4 years	186 months	23 (95.8%)	1 (4.2%)	2 (8.3%	-	-			Unknown
Khan *et al* 2005[22]	RCS	Type 2	C: Cast WB/NWB	**Total:** 31<br>14	14	**Total:** 20/12	**Total:** 28 years	16 months (range: 9 - 18)	8 (57.1%)	6 (42.9%)	-	-	-			Unknown
Kim *et al* 2017[23]	RC	Zone 1	S: IMS	**Total:** 29	**Total:** 30<br>15	6/9	47 years (range: 21 - 70)	**Total:** 13 months (range 12 - 15)	15 (100%)	0 (0%)	-	7.7 weeks (SD: 1.3, range: 5.9- 9.7)	100%		97.7 (SD: 3.4)	Unknown
			S: plate		15	5/10	50 years (range: 21 - 77)		15 (100%)	0 (0%)	-	5.93 weeks (SD: 1, range: 4.29- 7.29)	100%		98.2 (SD: 3.2)	
Konkel *et al* 2005[25]	RCS	Zone 1	C: mixed	**Total:** 46	**Total:** 48<br>35	6/27	38.3 years	**Total:** 32.4 months	25 (71.4%)	10 (28.6%)	-	16.1 weeks	-	17.4 weeks		Unknown
		Zone 2	C: mixed		10	3/7	48.6 years		8 (80%)	2 (20%)	-	15.2 weeks	-	15.2 weeks		
Koslowsky *et al* 2010[26]	RCS	Zone 1	S: k-wire	32	32	10/22	48.4 years (range: 24 - 88)	30.6 months (range: 15 - 60)	31 (96.9%)	1 (3.1%)	-	20.9 weeks	100%		96.5	53%
Lombardi *et al* 2004[30]	RCS	Zone 2	S: external-fixator	10	10	9/1	25.2 years (range: 15 - 49)	46 months (range: 14 - 79)	9 (90%)	1 (10%)	1 (10%)	6.5 weeks (SD: 0.5)	100%			90%

Study	Design	Zone	Treatment	No. patients	No. Fractures	Male/ female	Mean Age (yrs)	Mean FU (months)	Bone healing outcomes					Mean time return to activities/ work	Mean AOFA-score/ PROMS	Total % athletes
									Union (n, %)	Delayed/non-union (n, %)	Refracture (n, %)	Time to union (wks)	Displacement in % (>2mm)			
Mahajan *et al* 2011[32]	RCS	Zone 1	S: bi-cortical screw	**Total:** 23<br>6	**Total:** 23<br>6	2/4	47.2 years (range: 31 - 54)	**Total:**<br>22.5 months	6 (100%)	0 (0%)	-	5.3 weeks (SD: 1.0)	100%		93.5 (SD: 2.3)	Unknown
		Zone 2	S: bi-cortical screw	17	17	10/7	43.2 years (range: 16 - 74)		17 (100%)	0 (0%)	-	6.6 weeks (SD: 1.8)	100%		94.2 (SD: 2.4)	
Marecek *et al* 2016[34]	RCS	Zone 2	C: Shoe, WB	25	25	6/21	50.8 years[a] (range: 25- 80)		13 (52%)	12 (48%)	-	8.0 weeks (SD: 2.6)	-			Unknown
Mologne *et al* 2005[36]	RCT	Zone 2	C: Cast	**Total:** 37<br>18	**Total:**37<br>18	**Total:**<br>35/2	**Total:**<br>25.6 years (range: 18 - 58)	**Total:**<br>25.3 months (range: 15 - 42)	12 (66.7%)	6 (33.3%)	2 (11.1%)	14.5 weeks (SD: 4.7, range: 8 - 22)	-			100%
			S: IMS	19	19				18 (94.7%)	1 (5.3%)	-	6.9 weeks (SD: 2.3, range: 4-11)	-			
Monteban et al 2017[37]	RCS	Zone 1	C: U	**Total**: 93<br>72	72	33/39	37.9 years	25.0 months	69 (93.8%)	3 (4.2%)	-	-	29.2%		FFI: 7.4<br>VAS: 0.8<br>SF-36 PH: 72.7<br>MH: 74.8	40.3%
			S: U	21	21	7/14	43.7 years	37.7 months	20 (95.2%)	1 (4.8%)	-	-	76.2%		FFI: 7.3<br>VAS: 1.1<br>SF-36 PH: 63.4<br>MH: 84	61.9%
		Zone 2	C: U	**Total:** 59<br>49	49	17/32	59.4 years	27.8 months	49 (100%)	0 (0%)	-	-	12.2%		FFI: 8.5<br>VAS: 1.1<br>SF-36 PH: 65.2<br>MH: 77.7	36.7%
			S: U	10	10	6/4	54.7 years	29.5 months	10 (100%)	0 (0%)	-	-	60.0%		FFI: 6.8<br>VAS: 0.6<br>SF-36 PH: 79.3<br>MH: 84	40%
Portland *et al* 2003[42]	RCS	Zone 2	S: IMS	15	15		24 years	Total: 21 months (range: 6 – 52)	15 (100%)	0 (0%)	-	6.3 weeks	-			13.3%
Seitz *et al* 1985[46]	RCS	Type 2	C: cast	Total: 36<br>16	Total: 36	Total:<br>13/23	35 years (range: 11– 78)	To union	16 (100%)	0 (0%)	-		-			0%
			C: dressing	20					20 (90%)	2 (10%)	-		-			

Study [ref]	Design	Zone	Treatment	No. patients	No. Fractures	Male/ female	Mean Age (yrs)	Mean FU (months)	Bone healing outcomes					Mean time return to activities/ work	Mean AOFA-score/ PROMS	Total % athletes
									**Union (n, %)**	**Delayed/non-union (n, %)**	**Refracute (n, %)**	**Time to union (wks)**	**Displacement in %** (>2mm)			
Vorlat *et al* 2006[55]	RCS	Zone 1	C: Plaster	**Total:** 38<br>32	**Total:** 38<br>32	**Total:**<br>11/27	**Total:**<br>48 years<br>(range: 15 –78)	**Total:**<br>16.1 months<br>(range: 3.4- 30.9)	32 (100%)	0 (0%)	-	-	0%			Unknown
		Zone 2	C: Plaster NWB	6	6				3 (50%)	3 (50%)	-	-	0%			
Waverly *et al* 2018[56]	RCS	Zone 2	S: IMS	31	31	24/7	37.5 years<br>(SD: 12.6, range: 19 - 61)	18.6 months<br>(SD: 5.7, range: 12 - 24)	31 (100%)	0 (0%)	-	5.7 weeks<br>(SD: 1.5, range: 4 - 10)	-			Unknown
Shahdid *et al* 2013[47]	PC	Zone 1	C: cast	23	23	6/17	51.5 years	**Total:** 2.8 months						5.6 weeks		Unknown
			C: shoe	16	16	7/9	47.9 years							4.5 weeks		
Torg *et al* 1984[53]	RCS	Zone 2	C: Shoe, WB	**Total:** 43	**Total:** 46<br>10	**Total:**<br>41/2	**Total:**<br>18.6 years<br>(range: 14– 29)	**Total:**<br>40.2 months<br>(range: 6 – 108)	0 (0%)	10 (100%)	-	-	-			Unknown
			C: Plaster, NWB		15				14 (93.3%)	1 (6.7%)	-	7.4 weeks	-			
Tu *et al* 2017[54]	RCS	Zone 2	S: IMS	40	41	40/0		27 months<br>(SD: 22.3)	38 (92%)	3 (8%)	1 (2.4%)	-	-			100%
Van Aaken *et al* 2012[1]	PCS	Zone 1	C: bandage	**Total:** 23<br>15	**Total:** 23<br>15	3/12	47.5 years<br>(range: 16 - 74)	2.8 months	15 (100%)	0 (0%)	-	7.1 weeks<br>(range: 4- 12)	-		Modified foot score: excellent	Unknown
		Zone 2	C: bandage	8	8	2/6			8 (100%)	0 (0%)	-	7.3 weeks<br>(range: 4- 12)	-		excellent	
Wiener *et al* 1997[57]	PC	Zone 1	C: Cast	**Total:** 60<br>30	**Total:** 60<br>30	17/13	39 years	**Total:**<br>2.8 months	30 (100%)	0 (0%)	-	6.1 weeks	-		Modified foot score: 86	Unknown
			C: Dressing	30	30	14/16	36 years		30 (100%)	0 (0%)	-	6.4 weeks	-		92	

Study [ref]	Design	Zone	Treatment	No. patients	No. Fractures	Male/ female	Mean Age (yrs)	Mean FU (months)	Bone healing outcomes					Mean time return to activities/ work	Mean AOFA-score/ PROMS	Total % athletes
									**Union (n, %)**	**Delayed/non-union (n, %)**	**Refracute (n, %)**	**Time to union (wks)**	**Displacement in %** (>2mm)			
Wu *et al* 2017[58]	PC	Zone 1	S: IMS	**Total:** 41<br>21	**Total:** 41<br>21	13/8	25.6 years (SD: 7)	**Total:**<br>12 months	21 (100%)	0 (0%)	-		100%	8.1 weeks (SD: 0.9)	**AOFAS:** 88.1 (SD: 3) **VAS:** 0.4 (SD: 0.5)	100%
			C: Plaster	20	20	13/7	28.7 years (SD: 7.5)		17 (85%)	3 (15%)	-		100%	9.3 weeks (SD: 1)	**AOFAS:** 87 (SD: 3.7) **VAS:** 1.1 (SD: 1)	
Xie *et al* 2017[59]	RC	Zone 1	S: IMS	**Total:** 42	**Total:** 43<br>25	10/15	39.9 years	**Total:**<br>12 months	22 (88%)	3 (12%)	-	9.6 weeks (SD: 0.6)	100%	12.5 weeks (SD: 0.4)	87.8 (SD: 0.2)	Unknown
			S: Plate		18	5/13	34.4 years		16 (88.8%)	2 (11.2%)	-	7.5 weeks (SD: 0.1)	100%	12 weeks (SD: 0.5)	93.6 (SD: 0.3)	
Yoho *et al* 2015[61]	RCS	Zone 2	S: IMS	14	14	10/4	39.4 years (SD: 13.4, range: 17 - 61)	Max 12 weeks?	14 (100%)	0 (0%)	-	6.7 weeks (SD: 1.9, range: 4 – 9.1)	-			Unknown
Zhao *et al* 2017[63]	RCS	Zone 1	S: Plate	21	21	10/11	47.3 year (range: 27 –59)	14.3 months (range: 13- 19)	21 (100%)	0 (0%)	-	8.8 weeks (range: 7.4 – 12.1)	62%	11.4 weeks (range: 8.4 – 13)	93.2 (range: 87-100)	Unknown

a) transformed median to mean value
Abbreviations:
RCT: randomized controlled trial, PC: prospective comparative, PCS: prospective case series, RC: retrospective comparative, RCS: retrospective comparative, S: surgery, C: conservative, IMS: intramedullary screw, WB: weightbearing, NWB: non-weightbearing, U: unknown

Supplementary materials 2: search strategy

PubMed	EMBASE		CINAHL		Web of Science	
(diaphyseal[Title/Abstract] OR jone*[tiab] OR avulsion*[Title/Abstract] OR "Fractures, Bone"[Mesh] OR fracture*[tiab]) AND (("Metatarsal Bones"[Mesh] OR metatars*[tiab]) AND (5th[tiab] OR fifth[tiab] OR V[tiab])) OR "jones fracture"[tiab] OR "jones fractures"[tiab]	**#1**	jones fracture'/exp	S1	(MH "Jones Fracture")	**#1**	TOPIC: ("jones fracture" OR "jones fractures") Timespan: All years. Indexes: SCI-EXPANDED, SSCI, A&HCI, CPCI-S, CPCI-SSH, BKCI-S, BKCI-SSH, ESCI, CCR-EXPANDED, IC.
	**#2**	jones fracture*':ab,ti	S2	TI 'jones fracture*' OR AB 'jones fracture*'	**#2**	TOPIC: (diaphyseal OR jone* OR avulsion* OR fracture*)
	**#3**	fifth metatarsal fracture'/exp	S3	S1 OR S2	**#3**	TOPIC: (metatars* AND (5th OR fifth OR V))
	**#4**	#1 OR #2 OR #3	S4	TI metatars* OR AB metatars*	**#4**	#3 AND #2
	**#5**	metatars*:ab,ti	S5	TI (5th OR fifth OR V) OR AB (5th OR fifth OR V)	**#5**	#4 OR #1
	**#6**	fifth metatarsal bone'/exp	S6	(MH "Metatarsal Bones")		
	**#7**	metatarsal bone'/exp	S7	S4 OR S6		
	**#8**	#6 OR #7	S8	S5 AND S7		
	**#9**	(5th OR fifth OR V):ab,ti	S9	(MH "Metatarsal Fractures+")		
	**#10**	#8 AND #9	S10	TI ((diaphyseal OR jone* OR avulsion* OR fracture*)) OR AB ((diaphyseal OR jone* OR avulsion* OR fracture*))		
	**#11**	#5 OR #10	S11	S9 OR S10		
	**#12**	fracture'/exp	S12	S8 AND S11		
	**#13**	(diaphyseal OR jone* OR avulsion* OR fracture*):ab,ti	S13	S3 OR S12		
	**#14**	#12 OR #13				
	**#15**	#11 AND #14				

Supplementary materials 3 Clinical scoring systems reported in included studies ordered by number of appearances

Clinical scoring system	No. studies[a]
AOFAS Midfoot Score[24]	10
VAS Foot and Ankle[45]	4
Modified Foot Score[57]	2
AAOS Foot and Ankle Score	1
Olerud and Molander scoring system[38]	1
SFMA[51]	1
FFI[2]	1
SF12v2 (shorter version of SF-36[50])	1
SF-36[50]	1
None	15

a) articles included multiple scoring systems

Supplementary materials 4: Minors evaluation of included articles

	MINORS								Additional criteria for comaparative studies				
Study	A clearly stated aim	Inclusion of consecutive patients	Prospective collection of data	Endpoint appropiate to the aim of the study	Unbiased assesment of the study endpoint	Follow up period appropiate to the aim of the study	Lost of follow up less than 5%	Prospective calculation of study size	An adequate control group	Contampory group	Baseline equivalent of groups	Adequate statistical analysis	Total
Baumbach et al 2017	2	0	0	2	0	2	0	0	-	-	-	-	6/16
Biz et al 2018	2	1	0	1	0	2	0	0	1	2	0	2	11/24
Chee-kid et al 2009	0	2	0	1	0	0	0	0	-	-	-	-	3/16
Choi et al 2013	2	0	1	1	0	2	2	0	-	-	-	-	8/16
Chuckpaiwong et al 2008	2	1	0	1	0	2	0	0	1	2	1	1	11/24
Egol et al 2007	2	2	1	1	0	2	1	0	-	-	-	-	9/16
Ekstrand et al 2013	2	2	2	1	1	2	2	0	-	-	-	-	12/16
Gray et al 2008	2	0	2	0	0	0	0	0	1	2	1	2	12/24
Herrera-soto et al 2007	2	1	0	1	0	1	0	0	-	-	-	-	5/16
Josefsson et al 1994	0	1	2	1	0	2	0	0	-	-	-	-	6/16
Khan et al 2005	1	2	0	2	1	2	0	0	-	-	-	-	10/16
Kim et al 2017	1	2	0	2	1	2	0	0	2	2	0	2	14/24
Konkel et al 2005	0	2	0	1	1	2	0	0	-	-	-	-	6/16
Koslowsky et al 2010		1	1	0	2	2	0	0	-	-	-	-	8/16
Lombardi et al 2010	2	2	0	2	1	1	0	0	-	-	-	-	8/16
Mahajan et al 2011	1	1	0	2	1	1	0	0	-	-	-	-	6/16
Maracek et al 2016	1	2	0	2	1	0	0	0	-	-	-	-	6/16
Mologne et al 2005	2	2	2	2	2	2	1	0	2	2	2	2	21/24
Monteban et al 2017	2	1	0	1	1	2	0	0	-	-	-	-	7/16
Portland et al 2003	1	2	0	2	1	2	0	0	-	-	-	-	8/16
Seitz et al 1985	0	1	0	1	1	0	0	0	-	-	-	-	3/16
Vorlat et al 2006	1	1	0	1	1	2	1	0	-	-	-	-	7/16
Waverly et al 2018	2	1	0	2	2	2	0	0	-	-	-	-	9/16
Shadid et al 2013	2	2	2	2	1	1	1	0	2	2	1	1	17/24
Torg et al 1984	0	1	0	1	1	2	1	0	-	-	-	-	6/16

Tu et al 2017	2	1	2	2	2	0	0	0	-	-	-	-	9/16
Van Aaken et al 2012	1	1	2	1	1	2	0	0	-	-	-	-	8/16
Wiener et al 1997	2	2	2	2	1	1	1	0	2	2	1	1	17/24
Wu et al 2017	1	2	2	2	1	2	2	0	2	2	2	1	19/24
Xie et al 2017	1	2	0	2	1	2	2	0	2	2	1	2	19/24
Yoho et al 2015	2	1	0	2	1	1	0	0	-	-	-	-	7/16
Zhoa et al 2017	2	1	0	2	1	2	0	0	-	-	-	-	8/16
